# P53 and Beta-Catenin Activity during Estrogen treatment of Osteoblasts

**DOI:** 10.1186/1475-2867-5-24

**Published:** 2005-07-29

**Authors:** Nalini Chandar, Rasleen Saluja, Peter C Lamar, Kevin Kolman, Walter C Prozialeck

**Affiliations:** 1 Department of Biochemistry, Chicago College of Osteopathic Medicine, Midwestern University, 555, 31^st ^Street, Downers Grove, IL 60515, USA; 2 Department of Pharmacology, Chicago College of Osteopathic Medicine, Midwestern University, 555, 31^st ^Street, Downers Grove, IL 60515, USA

## Abstract

**Background:**

This study was undertaken to examine the relationship between the tumor suppressor gene p53 and the nuclear signaling protein beta-catenin during bone differentiation. Cross talk between p53 and beta-catenin pathways has been demonstrated and is important during tumorigenesis and DNA damage, where deregulation of beta catenin activates p53. In this study, we used estrogen treatment of osteoblasts as a paradigm to study the relationship between the two proteins during osteoblast differentiation.

**Results:**

We exposed osteoblast-like ROS17/2.8 cells to 17-beta estradiol (E2), in a short term assay, and studied the cellular distribution and expression of beta-catenin. We found beta-catenin to be up regulated several fold following E2 treatment. Levels of p53 and its functional activity mirrored the quantitative changes seen in beta-catenin. Alkaline phosphatase, an early marker of osteoblast differentiation, was increased in a manner similar to beta-catenin and p53. In order to determine if there was a direct relationship between alkaline phosphatase expression and beta-catenin, we used two different approaches. In the first approach, treatment with LiCl, which is known to activate beta-catenin, caused a several fold increase in alkaline phosphatase activity. In the second approach, transient transfection of wild type beta-catenin into osteoblasts increased alkaline phosphatase activity two fold over basal levels, showing that beta catenin expression can directly affect alkaline phosphatase expression. However increase in beta catenin activity was not associated with an increase in its signaling activity through TCF/LEF mediated transcription. Immunofluorescence analyses of p53 and beta-catenin localization showed that E2 first caused an increase in cytosolic beta-catenin followed by the accumulation of beta-catenin in the nucleus. Nuclear p53 localization was detected in several cells.

Expression of p53 was accompanied by distribution of beta-catenin to the cytoplasm and cell borders. A sub population of cells staining strongly for both proteins appeared to be apoptotic.

**Conclusion:**

These results suggest that interactions between p53 and beta-catenin signaling pathways may play a key role in osteoblast differentiation and maintenance of tissue homeostasis.

## Introduction

The organization of cells in tissues and organs is controlled by molecular control mechanisms that allow cells to interact with their neighboring cells and the extra cellular matrix. Cell-cell recognition and adhesion are critical processes in development, differentiation and the maintenance of tissue architecture. The cadherins family of Ca^2+^-dependent cells and their associated molecules such as beta-catenin are major components of the cellular adhesion machinery and play central roles in these various processes [1]. The cadherins are trans-membrane proteins that mediate Ca^2+ ^dependent cell-cell adhesion. Beta catenin is a multifunctional protein which associates with the intracellular domain of cadherins. In addition to providing a physical link between cells, these adherent junctional proteins influence various signaling pathways. Beta-catenin is an important component of the Wnt / Wingless signaling pathway and can act as a transcription factor in the nucleus by serving as a co activator of the lymphoid enhancer factor (LEF)/TCF family of DNA-binding proteins [2].

The p53 tumor suppressor gene acts as a guardian of the genome and a loss of its function is seen in a wider variety of cancers [3]. P53 acts by sensing DNA damage and directing the cell to arrest or undergo apoptosis [1,3]. In this way, p53 is thought to prevent the excessive accumulation of mutations that could give rise to malignancies. However, p53 activities may not be limited to tumor suppressor functions. Accumulating evidence suggests that p53 function may be critical during differentiation of various tissues and organs [4-7]. Defects in p53-null embryos have been reported, suggesting that p53 may have a role in tissue organization during development [8,9]. We have, in previous studies, demonstrated a role for p53 in osteoblast differentiation and expression of the bone specific protein osteocalcin [10]. In studies with p53 null and heterozygous mice, we have also shown that a decrease in p53 expression interferes with the ability of osteoblasts to express osteocalcin [11]. During in vitro osteoblast differentiation, proliferation is followed by matrix deposition and mineralization. Alkaline phosphatase is generally seen as an early marker of osteoblast differentiation, while osteocalcin is considered a late marker. In our studies with estrogen, we have shown p53 to be up regulated and its activity to be associated with cell cycle arrest and expression of osteoblast differentiation markers rather than apoptosis [12,13].

Cross talk between p53 and beta-catenin pathways has been demonstrated and appears to be especially important during tumorigenesis and DNA damage, where deregulation of beta catenin is known to activate p53 [14,15]. Because of the importance of the cadherins and beta-catenin in tissue differentiation, we wanted to determine if this type of cross talk with p53 exists in osteoblasts under physiological conditions. We observed expression of several apoptosis-related and cell cycle arrest proteins during short term treatment of bone cells with estrogen [13]. Expression of several caspases have been shown to be required for expression of bone markers during osteoblast differentiation [16]. Treatment with 17-beta estradiol did not result in any appreciable apoptotic cell death [12]. In studies reported here, we investigated if 17-beta estradiol could modulate the expression and subcellular distribution of beta catenin and how it might relate to p53 expression.

## Results

### 17-Beta estradiol (E2) up regulates expression of beta-catenin in osteoblastic osteosarcoma cells

**Figure 1:**
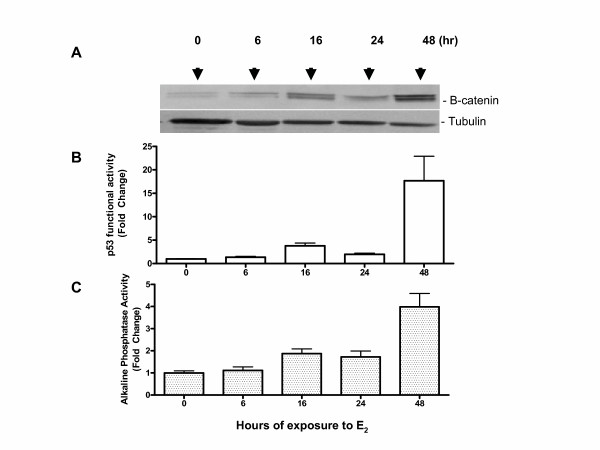
**(A)** 17-beta-estradiol treatment increases the amount of beta-catenin in ROS-PG13 cells: Osteoblasts were treated with 10^-11 ^M E2 for the times indicated and harvested for protein. The resulting protein lysates were subjected to western blot analysis of beta-catenin protein and beta-tubulin to monitor equal loading. The intensity of the bands was analyzed using a phosphorimager and results were normalized to beta-tubulin (loading control). A representative blot is shown. **(B)** Increase in p53 transactivating activity mirrors changes in beta-catenin. Equal amounts of E2 treated protein lysates described above were also subjected to a CAT assay to measure functional activity of endogenous p53 as described under methods. The resulting activity was plotted as fold change when compared to zero time (no treatment). The results represent mean ± SEM of 3 independent experiments in triplicates. **(C)** Alkaline phosphatase activity during E2 treatment: Enzyme activity was measured using a colorimetric assay as described under methods using the protein lysate described above. Values represent fold change when compared to control. Values represent mean ± SEM with an n of 3.

ROS17/2.8 cells stably expressing 13 copies of a p53 binding sequence (ROS-PG13CAT) fused to a chloramphenicol acetyl transferase (CAT) gene were used to study effects of estrogen on changes in endogenous p53 functional activity. Binding of endogenous p53 to the PG-13CAT sequence and subsequent activation of gene expression was studied by analyzing CAT activity as described in previous studies [17]. In all other aspects this cell line is representative of ROS 17/2.8 cells an osteoblastic osteosarcoma line that is used extensively to study osteoblast differentiation. These cells were treated with E2 for different lengths of time as described under Methods and the resultant protein was separated on SDS PAGE and analyzed by western blotting. As may be seen in Figure 1A, an increase in beta-catenin expression occurred within 6 h of treatment and peaked at 16 h of E2 treatment followed by a drop and a second peak during 48 h after E2 treatment. The first increase was less dramatic than the second increase in beta-catenin (10 vs 30 fold increase).

### P53 functional activity parallels changes in beta-catenin expression during E2 treatment

P53 function was monitored by measuring CAT activity in ROS-PG-13 cells. As may be seen in Figure 1B, p53 transcription activating activity was increased about 4-fold 16 h after E2 treatment followed by a drop and an increase corresponding to the change seen in beta-catenin at 48 h interval (about 17-fold). P53 expression is known to accompany beta-catenin activation and is also thought to be critical in the regulation of beta catenin function [15]. P53 expression was also measured by western blot analysis and was found to be high after 16 h and remained high until 48 h of E2 treatment (not shown).

**Figure 2:**
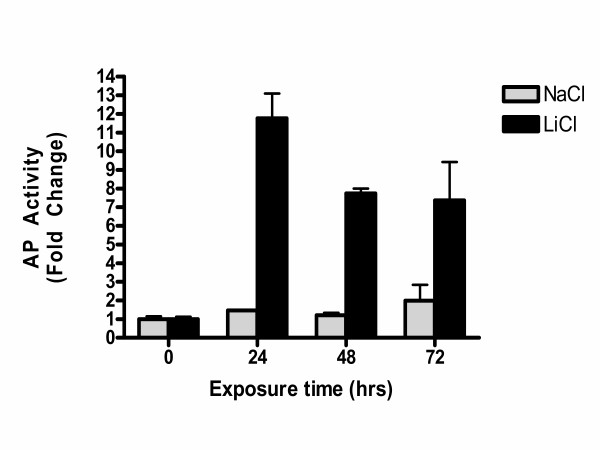
LiCl treatment of ROS-PG13 cells leads to activation of alkaline phosphatase expression. Protein lysates were prepared from cells treated with 10 mM LiCl or 10 mM NaCl and harvested after the times indicated. Equal amounts of protein extracts were used to measure alkaline phosphatase (AP) activity using p-nitro phenol phosphate as substrate at 420 nm and the relative activity of LiCl treated samples to NaCl treated controls is shown. The experiment represents mean ± SEM. n = 3 per treatment group.

### Alkaline Phosphatase, an early marker of bone differentiation is increased during treatment with 17-B-estradiol

Alkaline phosphatase activity was measured during the same time intervals using a colorimetric assay. While alkaline phosphatase increased steadily with E2 treatment, the enzyme activity showed a clear spike during the 48 h interval (Figure 1C). While initial induction of alkaline phosphatase activity occurred with an increase in beta-catenin activity, the subsequent boost to its activity was seen during 48 h corresponding to the large increase in beta-catenin activity.

**Figure 3:**
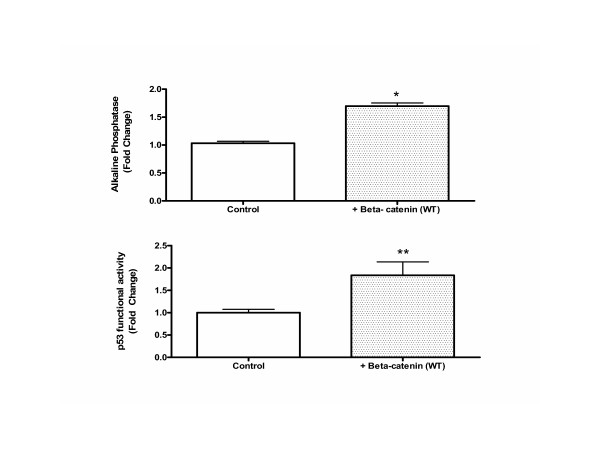
**(A)** Transient transfection of wild type beta-catenin into ROS-PG-13 cells increases alkaline phosphatase and p53 functional activity. Following transfection with the beta-catenin or control DNA, cells were lysed and equal amount of protein lysates were used to measure alkaline phosphatase activity. Enzyme activity was normalized to the mock transfected controls and reported as fold change. Data represents mean ± SEM of n = 4. *p < 0.05 versus control. **(B)** Transient transfection of wild type beta-catenin into ROS-PG-13 cells increases alkaline phosphatase and p53 functional activity. Lysates from the above experiment was also used to measure p53 activity by CAT assay. Equal amounts of protein were used and the resulting CAT activity is reported as a fold change over control levels. These experiments represent mean ± SEM of n = 4 ** p < 0.05 versus control.

### Is there a direct relationship between beta-catenin expression and alkaline phosphatase activity?

In order to determine if an increase in beta-catenin nuclear signaling activity is associated with increased alkaline phosphatase activity, we used a LiCl treatment as a model for beta-catenin activation. Treatment with LiCl is known to inhibit GSK activity, which is critical for phosphorylation and inactivation of beta-catenin function [18]. Immunofluorescent staining for beta-catenin revealed a transient increase in beta-catenin expression in the nuclei of ROS-PG-13 in 24 h 10 mM LiCl treated cells but not in the control NaCl treated cells (not shown). Protein lysates from the cells similarly treated with either LiCl or NaCl were tested for alkaline phosphatase activity. As may be seen in Figure 2, LiCl treated cells showed an increase in alkaline phosphatase activity 24 h after treatment, compared to a less than 2-fold activation in the NaCl treated cells (Figure 2).

**Figure 4:**
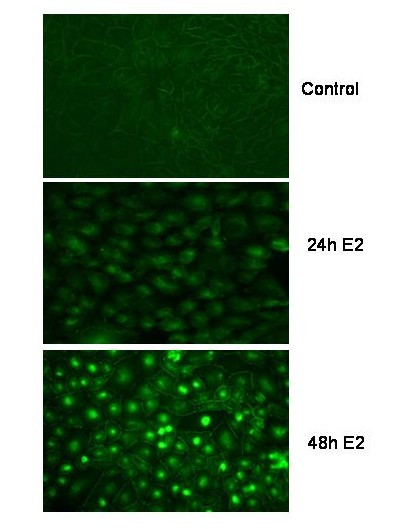
Beta-catenin expression during E2 treatment: Immunostaining of E2 treated ROS-PG13 with anti-beta-catenin antibody demonstrates its localization within the cells at 24 and 48 h after treatment. Cells were grown on cover slips and treated with 10^-11 ^M 17-beta estradiol in 2% charcoal treated serum containing media for the different lengths of time indicated. Control cells were grown in 2% charcoal treated serum containing media. Other details are as described under methods.

### Transient overexpression of wild type beta-catenin in ROS-PG13 cells increases alkaline phosphatase activity as well as p53 transcriptional activity

In order to determine if over-expression of beta-catenin produced similar effects on alkaline phosphatase, we transiently transfected a wild type beta-catenin plasmid into ROS-PG13 cells. Control cells were transfected with non-specific DNA. Alkaline phosphatase activity was measured in the control, mock-transfected and beta-catenin-transfected cells 24 h later. There was a small but statistically significant increase in alkaline phosphatase activity in beta catenin transfected cells when compared to cells that received non-specific DNA (Figure 3A). The same experiment was also repeated with a constitutively active beta-catenin (B-catenin S33Y) and similar results were obtained (not shown) suggesting that beta-catenin expression facilitates alkaline phosphatase expression in rat osteoblasts.

**Figure 5:**
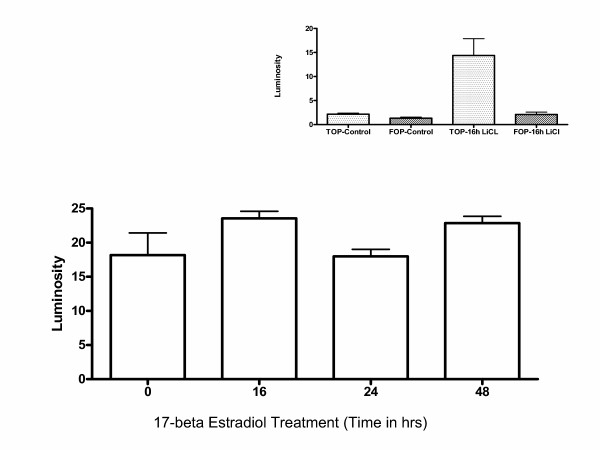
Increase in beta-catenin expression during E2 treatment does not result in beta-catenin signaling through TCF/LEF response elements in ROS-PG13 cells. Cells were transfected with TopFlash (wild type promoter) and FopFlash (mutant promoter) luciferase reporters in 2% media, and E2 treatment was started three hours later. E2 treatments were staggered during the 48 h interval and all cells were harvested after 48 h after the indicated time of exposure to the hormone. Mutant FopFlash activity was unchanged during the treatment and is not shown. LiCl treatment was carried out to demonstrate the validity of the assay (Inset). Cells were exposed to LiCl or NaCl 24 h after transfection for 16 h. In both these experiments luciferase activity was measured in cell lysates using equal amounts of protein. Experiments represent average ± SEM of triplicate measurements.

Protein lysates from the transiently transfected cells were subjected to CAT assay for determination of p53 functional activity during the same time period. P53 activity was 5 fold higher in cells transfected with wild type beta-catenin when compared to control cells (Figure 3B), showing that a parallel increase in p53 activity may not be limited to conditions of DNA damage but also occurs under physiological conditions.

**Figure 6:**
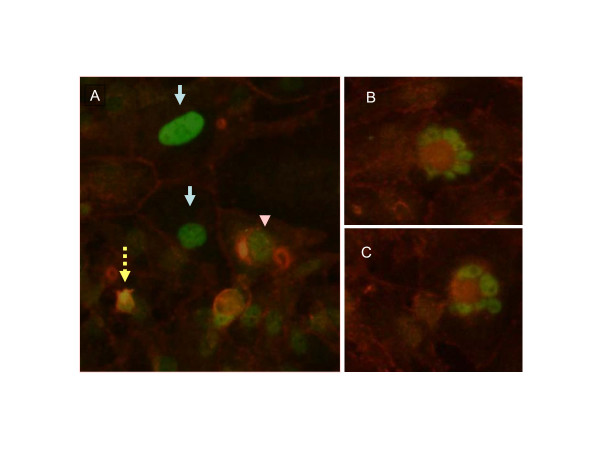
Immunohistochemical staining of beta-catenin and p53. Cells were treated with 10^-11 ^M 17-beta estradiol for 48 h as described under methods and stained for p53 (green) and beta-catenin (red) using specific antibodies. Panel A shows cells strongly staining for both proteins in the nucleus (broken arrow), strong p53 staining in the nucleus and beta-catenin at the plasma membrane (solid arrows) and strong staining of p53 in the nucleus with beta-catenin relegated to the cytoplasm (arrow head). B and C show apoptotic cells with strong staining of both proteins.

### Subcellular distribution of beta-catenin during treatment

In order to determine the localization of beta-catenin during the treatment protocol, we conducted immunofluorescence analyses of estrogen treated cells (Figure 4). Cells were grown to confluency and switched to 2% charcoal treated media for 24 h before exposure to 17-beta estradiol. At the start of experiment (0 time), beta-catenin staining was only seen at the adherent junctions between cells and was undetectable intracellularly. 24 h after treatment with 17-beta estradiol, there was a dramatic increase in the amount of beta-catenin within the cells; most of the beta-catenin appeared to be in the cytoplasm and peri nuclear region. By 48 h strong staining for beta-catenin could be detected within the nucleus of a significant number of cells.

### No change in beta-catenin transcriptional activity during E2 treatment

Since we observed nuclear staining of beta-catenin, experiments were carried out to determine if beta-catenin signaling through TCF/LEF family of transcriptional factors was activated. We transiently transfected the wild type TCF/LEF response elements (TOPFLASH) or the mutant sequence (FOPFLASH) followed by treatment with E2 treatment. No significant change in luciferase activity was noted during E2 treatment (Figure 5). The validity of the assay was checked using LiCL treatments (Figure 5 insert). These results indicate that endogenous beta-catenin signaling is not activated during E2 treatment even though the expression of beta-catenin was observed in the nuclei of treated cells.

### p53 expression during 17-beta estradiol treatment

The patterns of p53 distribution were also monitored by immunostaining. Immunofluorescence staining for p53 also showed a heterogeneous pattern. P53 expression was high within the nucleus in a number of isolated cells. Among the cells that stained strongly for p53, some of them were apoptotic and counter staining with Hoescht reagent showed a pyknotic nucleus. In other cases strong staining was evident in nuclei that looked morphologically normal. P53's presence in the nucleus was also confirmed with western blots of cytoplasmic and nuclear fractions (not shown). Its presence in the nucleus correlated with its functional activity as measured by the CAT assay.

A better understanding of the relationship between the two proteins was evident when we stained simultaneously for both proteins and a representative field is shown in figure 6. Three types of association were evident. Strong staining of nuclear p53 was accompanied by beta-catenin in the cell borders (arrows). When both proteins were present in the nucleus, the cell was generally apoptotic (broken arrows). When intracellular staining for beta catenin was strong it was mostly contained in the cytoplasm when p53 decorated the nucleus (arrow head).

## Discussion

In previous studies, we have shown the tumor suppressor gene p53 to be up regulated by estrogen and to be important for differentiative functions in bone [12,13]. In the studies reported here, we show that beta-catenin expression is increased during estrogen treatment of osteoblasts. This large increase in beta-catenin expression that we observed may be the result of either a direct increase in gene expression, or from stabilization of cytosolic beta-catenin. With regard to the latter possibility it is worth noting that in other cell types, estrogen has been shown to inhibit GSK activity which results in the stabilization of beta-catenin [18].

The association of beta catenin activation with increases in alkaline phosphatase expression is also very interesting, but not completely new. This association has been recently detected in several cell types where alkaline phosphatase plays a role in differentiated behavior of the cell [19-21]. Recent studies have implicated the wnt signaling pathway and beta-catenin in the regulation of alkaline phosphase expression in osteoblasts [21]. It appears that beta-catenin is able to increase alkaline phosphatase albeit indirectly, because no TCF binding sites have been detected within the alkaline phosphatase gene [22].

The role of p53 in the regulation of beta-catenin is best understood under conditions of DNA damage and tumorigenesis [15]. Stabilization of beta-catenin has been observed to cause stabilization of p53 through inhibition of its degradation [14,23,24]. While it is possible that beta-catenin results in the stabilization of p53, the resulting increase in p53 is not responsible for apoptosis, an activity that is regulated by p53 during DNA damage. Instead, under physiological conditions, p53 appears to monitor the environment such that an abnormal increase in beta catenin within the nucleus results in apoptosis, while in other cells the presence of p53 in the nucleus prevents the accumulation of beta-catenin. Beta catenin under these conditions appears to be relegated to the plasma membrane.

In the studies reported here we show treatment with 17-beta estradiol increases expression of beta-catenin and cause its migration in to the nucleus. Estrogen may mediate this effect by its action on GSK activity as seen in other tissues [25]. However, beta-catenin expression in the nucleus does not result in the activation of its signaling through TCF/LEF transcription factor binding sites. There are several likely reasons for this observation. As has been noted earlier, the level of signaling through the canonical pathway may be low and below detection limits using TCF/LEF reporter constructs [26]. It is also possible that beta-catenin may not directly act through the Wnt canonical pathway, but crosstalk with other pathways to generate a response. It has been shown that beta-catenin signaling does not function independently but synergizes with morphogens like BMP-2 to induce the early bone phenotypes in undifferentiated cells [21,22]. In a similar manner, estrogen treatment has been observed to enhance the binding of beta-catenin to estrogen receptors alpha and beta in human colon and breast cancer cells [27] and also participate in the transactivation of estrogen responsive genes. This suggests that beta-catenin may function as a common mediator of different bone specific agents to induce early bone phenotype. In this context it is interesting that beta-catenin and LEF1 repress expression of the osteocalcin gene, a late marker of the bone phenotype [28].

While the role of estrogen as bone-protective anabolic agent is well established, the mechanism of action is only now being understood at the molecular level [29,30]. Estrogen affects osteoblasts by non genotropic mechanisms that go to increase the life span of the osteoblasts by its action on plasma membrane signaling proteins [31]. Antiapoptotic mechanism by estrogen is transient in osteoblasts and it is not clear if p53 plays a role in this process. In a manner similar to estrogen receptors, p53 has been shown to bind beta-catenin resulting in its stabilization and transcriptional activation [23]. P53 is also able to inhibit expression of TCF-4 by directly binding to the promoter of the gene [32]. This type of regulation may be important to maintain cell-cell interactions and prevent apoptosis. These types of cross signaling may be relevant and important for osteoblast differentiation as opposed to osteoblast proliferation and may critically depend on the cellular environment. P53 is known to interact with a plethora of proteins [33] and these interactions may determine the final outcome for the cell. P53's ability to sense the environment allows for cell cycle arrest and differentiation under some circumstances and apoptosis in other instances. Expression of alkaline phosphatase a differentiation marker in bone may be facilitated by beta-catenin nuclear activity. However once alkaline phosphatase is increased, p53 activity may be critical to maintain the differentiated behavior of the cell by making sure beta-catenin is retained at cell borders rather than within the nucleus. Further studies are required to understand how the interactions between estrogen receptors, beta-catenin, p53 and related proteins facilitate the differentiation process.

## Conclusion

Our data shows that beta-catenin activity is modulated during estrogen induced osteoblast differentiation and its increase is associated with an increase in p53 and alkaline phosphatase. The cellular localization of endogenous p53 and beta-catenin appears be mutually exclusive during estrogen treatment and reflects the role of p53 in regulating growth and differentiation.

## Methods

### Establishment of cell lines

The cell line ROS 17/2.8, a rat osteosarcoma cell line, was kindly provided by Dr. G. Rodan (Merck, Research Laboratory, West Point, PA). Cells were grown in minimal essential medium with α-F12 with 10% fetal bovine serum in a modified atmosphere of 95% air and 5% CO_2 _at 37°C. This cell line contains a wild type endogenous p53 [17] and can be induced to mineralize in culture and express genes associated with advanced stages of differentiation. The ROS17/2.8 cells were stably transfected with the plasmid PG-13-CAT (a kind gift of Dr. B. Vogelstein, Johns Hopkins University, Baltimore, MD). This plasmid encodes 13 copies of a p53 binding DNA sequence fused to a CAT reporter gene)[17]. In the present studies cells transfected with this plasmid (referred to as ROS-PG13) cells were used to monitor transcriptional activity of endogenous p53.

### Cell Culture conditions & Treatment with 17β-Estradiol

Cells for E2 treatment were exposed to phenol red free media before and during treatment with E2. The water-soluble form, 17β-estradiol (Sigma, St. Louis, MO) was used at the concentration of 10^-11 ^M. Cells used for E2 treatment were exposed to 2% charcoal-treated serum containing phenol red free media for 24 hours before treatment with E2. For experiments requiring E2 for longer than 24 hours, fresh media with E2 was maintained on cells. Unless otherwise mentioned, all experiments were done using E2 at a final concentration of 10^-11 ^M. This concentration is based on results obtained with our previous studies, where we saw maximal induction of p53 at 10^-11 ^M – 10^-12 ^M [12]. Cells were treated for different lengths of time ranging from 0–72 h.

### Transient Transfections

For beta-catenin transfections, we used HA-β-catenin (WT beta catenin) and S33Y β-catenin (a constitutively active mutant), a kind gift of Dr. Ben-Ze'ev, Weizmann Institute, Rehovot, Israel. Cells were transfected with Superfect (Qiagen) in 10-cm plates for 24–48 h followed by protein lysis. The total amount of DNA used was maintained equally in these experiments. Equal amount of protein was used for measurement of alkaline phosphatase and CAT activity.

### Measurement of CAT Activity

CAT activity of ROS-PG13 cells after treatment was used as a measure of p53 DNA binding activity and reflected p53 function at any time point. Harvested cells were suspended in buffered saline and then in a 0.25 M Tris buffer pH 7.8, disrupted by 3 freeze-thaw cycles. The supernatants were collected after centrifugation and heated at 65°C for 10 minutes to inactivate cellular acetylase activity. Protein concentrations were measured with the Bradford method and equal amounts of protein were used in the assays. CAT activity was determined by means of liquid scintillation counting, and was measured over a linear range of chloramphenicol acetylation such that the fraction acetylated was proportional to actual activity. All measurements were carried out on triplicate samples. Other details are as described earlier.

### Measurement of Luciferase Activity

For reporter assays, cells were transfected with the beta-catenin responsive firefly luciferase reporter plasmids TopFlash (wild type promoter) or FopFlash (Mutant promoter) (Upstate Biotechnologies) for 48 h. Three hours after transfection, cells received 17-beta estradiol to a concentration of 10–11 M for the times indicated. Cells were exposed to LiCl for 16 hours, lysed and equal amount of protein was used for measuring luciferase activity. All measurements were carried out on triplicate samples and experiments were repeated at least thrice.

### Immunofluorescence staining

Beta-catenin and p53 were visualized by indirect immunocytochemistry using a rabbit anti beta catenin (Zymed Laboratories Inc., San Francisco, CA.) or a mouse anti-p53 (1C12) (Cell Signaling Technology, Beverly, MA.) as the primary antibodies. ROS-PG13 cells were plated on coverslips and treated with E2 as described above. Cells were fixed in ice cold methanol and permeabilized for ten minutes. Cells were then blocked with 10% goat serum for 10 minutes room temperature. Samples were incubated for 1 hour with primary antibody followed by a-30 minute incubation with a goat, anti-rabbit TRITC-conjugate or goat, anti-mouse FITC-conjugate. Cells were then viewed with a Nikon Eclipse 400 fluorescence microscope using 40× and 100× objectives. Digital images were captured with a Spot digital camera (Diagnostic Instruments, Sterling Heights, MI) using automated exposure times and gain settings for the bright-field images. Dark-field fluorescence images were captured using a gain setting of 16 and exposure times of 3 s for green and 1 s for red and blue. The digital images were processed using the Image-Pro Plus images analysis software package (Media Cybernetics, Silver Spring, MD).

Negative controls consisted of samples that were incubated without the primary antibodies. All labeling experiments were repeated at least three times and were highly reproducible.

### Immuno Blotting

Protein lysates were prepared using M-PER Reagent (Pierce, Rockford, IL) combined with a protease inhibitor cocktail, Complete Mini (Roche, Mannheim, Germany). Twenty-five micrograms of each protein lysate was subjected to 10% SDS-PAGE, and transferred to immun-Blot PVDF membrane (Bio-Rad, Hercules, CA). Expression was determined using rabbit anti beta catenin (Cell Signaling Technology, MA) and HRP-goat anti rabbit conjugate (Zymed Laboratories Inc.CA). Membranes were then developed using enhanced chemiluminescence (Amersham International, Amersham, Bucks, UK).

### Alkaline Phosphastase

Alkaline phosphatase activity was measured using a quantitative colorimetric assay with para-nitrophenol phosphate as substrate using a commercially available kit (Sigma Chemical Company, St. Louis, MO).

### Statistical Analyses

The differences in the means of experimental results were analyzed for their statistical significance with the one-way ANOVA combined with a multiple comparison procedure (Tukey Kramer multiple comparisons test).
